# Physiological Chemistry

**Published:** 1905-11

**Authors:** John A. Miller

**Affiliations:** Buffalo, N. Y.


### PROGRESS IN MEDICAL SCIENCE.

### Physiological Chemistry.

BY JOHN A. MILLER, PH. D., Buffalo, N. Y.

MICRO-RESPIROMETRIC INVESTIGATIONS by Forsten Thunberg (Centr. Physiol., 18,—J. C. Soc., 88,44). The question whether nerves participate in respiratory activity has been answered in the affirmative by Baeyer, Fröhlich, and others, in confirmation of Waller's long-expressed opinion that carbon dioxide is produced during the activity of nerve fibers. By means of an apparatus termed the "micro-respirometer," the respiratory exchanges in small objects like nerves can be actually measured. A number of results are given.

---

INFLUENCE OF SURGICAL OPERATIONS ON CARBOHYDRATE METABOLISM, by Edward Pflüger, Bernhard Schöndorff, and Frederick Wenzel (Pflüger's Archiv., 105; J. Ch. Soc., 88, 44). Glycosuria is often described as a common sequel to surgical operations. The grounds for this opinion are not regarded as satisfactory; the urine may contain a reducing substance, but this is not necessarily sugar. A discussion follows concerning the best tests for sugar in urine. Even the fermentation test is untrustworthy. Most reliance should be placed on the polarimetric test and Worm-Müller's modification of the copper test. From the examination of some hundreds of urines, the conclusion is drawn that many forms of so-called transitory glycosuria (including those produced surgically and by anesthesia) do not exist.

---

Action of Salts on Muscle and Nerves, by Ernest Overton (Pflüger's Arch., 105, 176; J. C. Soc., 88, 46). The experiments were mainly made with thin muscles, like the frog's sartorius. Solutions of potassium chloride isotonic with blood kill the muscles in a few minutes and cause them to increase in weight. In a mixed solution of the chloride of potassium and sodium, the muscle remains almost impermeable to potassium chloride until it is injured by that salt. Other potassium haloids and potassium nitrate behave in the same way. Loss of indirect excitability occurs rapidly when quite a small percentage (0.06 to 0.07) of potassium chloride is added to a sodium chloride solution; this effect is removed by the addition of calcium chloride. The harmful action attributed to potassium ions is like that caused by curare. Rubidium, caesium and ammonium salts act like potassium, with some differences of detail. Strontium acts like calcium as an antagonist to these, but barium and magnesium salts do not. It can not, therefore, as Loeb supposes, be a simple question of valency of ions.

---

Biological Importance of Iron, by A. Baldwin (Arch. exp. Path. Pharm., 52,61; J. C. Soc., 88, 46). Iron in nutriment is not only of importance in hemoglobin formation, for all the tissues contain iron. The blood-free epidermis, the crystalline lens, the tissues of the crayfish, and, among plants, Iceland moss were investigated, and all were found to contain small amounts of iron.

---

Urinary Indoxyl, by Leon Grimbert (J. Phar. Chim., 1904; J. C. Soc., 88, 48). Human urine always contains indoxyl; its presence is not pathological; the amount varies with food, work, fatigue, and intestinal disorders. Even large quantities have no necessary importance; there is no ground for supposing it to be due to liver disease. Scatoxyl pigments do not occur in urine, for by oxidation in the body scatole loses its methyl group and is converted into indoxyl. Red colors attributed to scatoxyl are produced by indirubin.

STUDIES IN DIURESIS. THE SITUATION IN THE KIDNEY WHERE FOREIGN SUBSTANCES ARE EXCRETED, by Joh. Biberfeld (Pflüger's Arch., 105, 308; J. C. Soc., 88, 48). The experiments were made with pigments, especially Berlin-blue, and they lead to the conclusion that this substance is excreted by the convoluted tubules. This work confirms in the main von Soberierauski's recent researches with indigo-carmine. Some of the observations throw doubt on Ludwig's doctrine of reabsorption of water in the tubules.

---

ADRENALINE AND ALKYLAMINOACETYLCATECHOL, by F. Stolz, (Ber. 37, 4149; J. C. Soc., 88, 106). The author's results obtained by methylating and oxidising adrenaline are identical with those of Jowett. Methyl iodide and methyl-alcoholic sodium hydroxide react with adrenaline to form vanillin. These reactions indicate the formula of adrenaline as being $C_6 H_3 (OH)_3 CH(OH).CH_2.NHCH_3$., and it should therefore be possible to synthesise the base by reducing methylaminoacetylcatechol.

[With Hans Meyer (Marburg)] — The alkylaminoacetylcatechols (J. C. Soc., 1904, 1, 873) and their reduction products have physiological properties closely resembling those of adrenaline.

---

DECOMPOSITION OF IODOFORM BY THE ACTION OF OXYGEN AND LIGHT RAYS., by E. Van Aubel, (Chem. Centr. 1904, ii, 1376; J. C. Soc., 88, 1). Mixtures of iodoform with substances which are not liquid, such as vaseline, are decomposed by sunlight and by radium rays, but when exposed to moderate light in winter at—45°, no decomposition could be detected.

